# Complement factor H *Y402H* polymorphism results in diminishing CD4^+^ T cells and increasing C-reactive protein in plasma

**DOI:** 10.1038/s41598-023-46827-0

**Published:** 2023-11-08

**Authors:** Marie Krogh Nielsen, Yousif Subhi, Mads Falk, Amardeep Singh, Torben Lykke Sørensen, Mogens Holst Nissen, Carsten Faber

**Affiliations:** 1 Clinical Eye Research Division, Department of Ophthalmology, Zealand University Hospital, Roskilde, Denmark; 2 Department of Ophthalmology, Copenhagen University Hospital, Rigshospitalet, Denmark; 3 Department of Clinical Research, University of Southern Denmark, Odense, Denmark; 4 Department of Clinical Medicine, University of Copenhagen, Copenhagen, Denmark; 5 Department of Immunology and Microbiology, University of Copenhagen, Faculty of Health and Medical Sciences, Copenhagen, Denmark

**Keywords:** Complement cascade, Macular degeneration

## Abstract

Age-related macular degeneration (AMD) is a common cause of visual loss among the elderly. Genetic variants in the gene encoding complement factor H (CFH) have been identified as an AMD susceptibility gene, however, the mechanistic link is debated. Here, we investigated the link between the CFH Y402H genotype and low-grade inflammation. We recruited 153 healthy individuals, 84 participants with dry stages of AMD, and 148 participants with neovascular AMD. All participants were subjected to detailed retinal examination, and interview regarding comorbidities and lifestyle. Blood samples were analyzed for level of C-Reactive Protein (CRP), white blood cell differential count, and stained with fluorescent antibodies to differentiate CD4^+^ and CD8^+^ T cells. CFH Y402H genotyping was performed using an allele-specific polymerase chain reaction genotyping assay. Splenocytes from young and aged wild type and *Cfh* null mutant C57BL/6J mice were examined for CD4^+^ and CD8^+^ T cells. Healthy individuals with the CFH Y402H at-risk polymorphism HH had higher levels of CRP and lower proportions of CD4^+^ T cells compared to persons with the YH or YY polymorphism (P = 0.037, Chi-square). Healthy individuals with the HH polymorphism displayed lower proportions of CD4^+^ T cells with ageing (P < 0.01, one-way ANOVA), whereas both young and aged *Cfh* null mutant mice displayed lower proportions of CD4^+^ T cells (P < 0.001 and P < 0.05; unpaired t test). Participants with dry AMD and the HH polymorphism had similarly lower proportions of CD4^+^ T cells (P = 0.024, one-way ANOVA), but no difference in CRP-levels. In the neovascular stage of AMD, there was no difference in proportion of CD4^+^ cells or CRP levels according to genotype. The risk-associated CFH genotype is associated with an age-related decrease in proportion of CD4^+^ T cells and increased levels of CRP in healthy individuals. This indicates that decreased complement regulation results in extensive changes in innate and adaptive immune compartments that precede development of AMD.

## Introduction

Age-related macular degeneration (AMD) is a common debilitating retinal disease among the elderly, robbing them of visual function and quality of life. Recent research into the pathogenesis of AMD describes a complex disease mechanism with local and systemic inflammation. As such, nearly all parts of the immune system have been associated with AMD^1, 2^. Much focus has been drawn to the complement system because of a strong association between a small nucleotide polymorphism (SNP) in complement factor h (CFH) Y402H and development of AMD^3^. Several lines of research have also associated development of AMD with increased levels of CRP^4, 5^ as well as various phenotypically and functional alterations in circulating immune cells^6–11^.

CFH is a negative regulator of the complement system and CFH Y402H carriers have slightly increased activity in the complement system^12–14^. The complement is part of the innate immune system and works as a strong regulator of immune cells from both the innate and adaptive immune system^15^. The CD4^+^ T cells are a group of heterogeneous cells that play a pivotal role in the immune system through several surface receptors including complement receptors^16^. With age, various phenotypical and functional changes of the innate and adaptive arms of the immune system have been observed, a phenomenon known as immunosenescence. A relatively simple measure of immunosenescence is the decline of circulating CD4^+^ T cells with age^17, 18^.

Based on the increased complement activity and possible interactions with immune cells, we hypothesized that CFH Y402H carriers show signs of increased inflammation with ageing.

The purpose of the present study was to investigate how the CFH Y402H polymorphism alters markers of ageing and inflammation in a group of individuals with no AMD, in a murine model of CFH deficiency, and in individuals with AMD (Table 1).

**Table 1:** Study questions.

1.	Question	Does CFH polymorphism affect markers of inflammation and ageing in a population of healthy individuals?
	Population	153 participants with no retinal disease and less than 10 small drusen
2.	Question	Does CFH deficiency affect T cell distribution in mice?
	Population	Young (7–8 weeks of age) and aged (52–54 weeks of age) WT and *Cfh*^−/−^ C57BL/6J mice. Six mice in each group
3.	Question	Does CFH polymorphism affect markers of inflammation and ageing in a population with dry AMD?
	Population	84 participants with drusen maculopathy, changes in retinal pigment epithelium and in 59 (70%) cases also atrophic lesions
4.	Question	Does CFH polymorphism affect markers of inflammation and ageing in a population with neovascular AMD?
	Population	143 individuals with drusen, fibrovascular pigment epithelium detachment with angiographic leakage and exudative changes in the retina

All human participants were recruited in studies performed in 2010–2013 and in 2015–2018.

## Methods

### Design

This prospective study was approved by the Regional Committee of Ethics in Research of the Region of Zealand (SJ-385/SJ-142). Oral and written informed consent was obtained from all participants prior to inclusion after explanation of the nature and possible consequences of the study. The study adhered to the tenets of the Declaration of Helsinki.

This study was designed as a series of observational studies, with immunological data obtained during two separate cohort studies in the period 2010–2013 and in 2015–2018.

### Study participants and inclusion

Patients and their spouses attending the Department of Ophthalmology, Zealand University Hospital, were invited to participate. Individuals were not included if they had any cancer, infectious or autoimmune diseases, or if they received any immune-modulating medication. Participants underwent an interview regarding medical history, current medication use, height and weight, weekly alcohol consumption, and tobacco use.

### Retinal diagnosis

All participants were subjected to a detailed retinal examination, including dilated funduscopic examination, digital color fundus photography (Carl Zeiss, Germany), spectral-domain optical coherence tomography, and autofluorescence imaging (Heidelberg Engineering, Heidelberg, Germany). Fluorescein and indocyanine green angiography was performed in cases suspected of neovascular AMD to confirm the final diagnosis.

Individuals were categorized based on the Clinical Age-Related Maculopathy Grading System (CARMS) described by Seddon et al.^19^ into three groups: No AMD—less than 10 small drusen without pigment abnormalities corresponding to CARMS grade 1. Dry AMD—one or more of the following signs: Drusen maculopathy, pigment abnormalities, atrophic lesions corresponding to CARMS grade 2–4. Neovascular AMD—non-drusenoid pigment epithelial detachments, serous retinal detachments, choroidal neovascular membrane with angiographic leakage corresponding to CARMS grade 5. The highest CARMS grade received from both eyes was used for participant classification.

### Blood sampling and analysis

Fresh venous blood samples were obtained. One lithium-heparin-coated tube was used for measuring CRP (Dimension Vista 1500, Siemens Healthineers, Erlangen, Germany). One ethylenediaminetetraacetic acid (EDTA)-coated tube was used for automated white blood cell differential counts (Sysmex KX-21NTM, Sysmex Corporation, Kobe, Japan). Another EDTA-coated tube was used for genotyping purposes. Extraction of DNA was performed on EDTA-stabilized venous blood using Chemagic Magnetic Separation Module 1. The DNA samples were genotyped at LGC genomics, using the Kompetitive Allele Specific Polymerase chain reaction (KASP) genotyping assay. A third tube with EDTA-stabilized fresh venous blood was prepared for flow cytometric analysis. We used the differential count to obtain a fixed number of cells in the sample (500,000 leukocytes). The sample was lysed and washed using red blood cell lysis buffer (Nordic Biosite AB, Sweden). The sample was washed three times by centrifuging and resuspending in isotonic buffer. Fluorescent monoclonal antibodies were added to the test tube, and cells were incubated for 20 min in darkness. Antibodies used for CD4 were Peridinin-chlorophyll protein complex, IgG2a (FAB3791C-100, R&D Systems Inc., MN, USA), and for CD8 was Phycoerythrin-CY7, IgG1 (300914, BioLegend, CA, USA). After incubation, cells were washed and analyzed using flow cytometry (BD FACSCanto IITM, BD Biosciences, NJ, USA).

### Animal studies

We used young (7–8 weeks of age) and aged (52–54 weeks) WT and *Cfh*^−/−^ C57BL/6J mice. Six mice were in each group. WT mice purchased from Harlan Laboratories; *Cfh*^−/−^ mice provided by Matthew Pickering^20^. All animal experiments adhered to the ARVO Statement for the Use of Animals in Ophthalmic and Vision Research and were approved by the UCL Institute of Ophthalmology Ethical Review Panel.

Following cervical dislocation, spleens were removed and meshed to single-cell suspensions. Cells were washed and stained for flow cytometry as stated above with the following antibodies: CD3-pacific blue IgG2b (100213, BioLegend, CA, USA), CD4-APC IgG2a (100515, BioLegend, CA, USA) and CD8-APC-Cy7 IgG2a (301016, BioLegend, CA, USA). Samples were analyzed using flow cytometry (BD LSR II, BD Biosciences, NJ, USA).

### Data analysis and statistics

Participant characteristics were summarized in groups defined by stage of AMD. In each group, we compared demographics and measurements based on CFH Y402H polymorphism. We also included the calculated CD4/CD8 and neutrophil/lymphocyte ratios as these measures of immunosenescence and inflammation have been used by others^18, 21^. Statistical analysis was performed using the Statistical Package for the Social Sciences, SPSS version 25 (IBM Corporation, Armonk, NY, USA). Normally distributed data was presented using mean and standard deviation (SD) and comparisons were made using parametric tests. Not normally distributed data was presented using median and interquartile range (IQR) and comparisons were made using non-parametric tests. Categorical data was presented in percentage and tested using χ^2^ test, with the exception of tests with small categories (< 5 cases) in which case Fisher’s Exact test was used.

## Results

### Participants

A total of 380 participants were recruited for this study. Of these, 143 had neovascular AMD, 84 had dry AMD, and 153 were healthy individuals. The average age of all participants was 71 years (SD: 12.9), and 227 (59.7%) were female. The healthy individuals were younger than patients with AMD (mean age 62.5 years, SD: 14.3 vs. 76.8 years SD: 7.7). The at-risk SNP HH was more frequent among participants with neovascular AMD (40% of participants), compared to those with dry AMD (29.8% of participants), and those without AMD (13.1% of participants) (P < 0.001)***.*** The CFH Y402H SNP did not deviate from the Hardy-Weinbergs equilibrium in healthy individuals: (χ^2^ = 3.04, P = 0.10), participants with dry AMD (χ^2^ = 0.40, P = 0.53), or participants with neovascular AMD (χ^2^ = 0.52, P = 0.47).

The group of healthy individuals were defined as healthy based on the retinal analysis. While some of these individuals had hypertension, hyperlipidemia, cardiovascular disease, and/or type 2 diabetes, the frequency of systemic disease was the same across CFH Y402H genotypes (Table 2).

**Table 2:** Characteristics of the population of healthy individuals (CARMS 1) according to CFH Y402H genotype.

	CFH Y402H			P-value
	HH, N = 20	YH, N = 73	YY, N = 60	
Age, mean (SD)	63.9 (11.83)	64.7 (14.2)	59.4 (14.9)	0.096^a^
Gender				
F, n (%)	17 (85)	44 (60)	34 (57)	0.069^b^
M, n (%)	3 (15)	29 (40)	26 (43)	
Hypertension				
No, n (%)	14 (70)	50 (69)	50 (83)	0.136^b^
Yes, n (%)	6 (30)	23 (31)	10 (17)	
Hyperlipidemia				
No, n (%)	19 (95)	62 (85)	48 (80)	0.304^b^
Yes, n (%)	1 (5)	11 (15)	12 (20)	
Cardiovascular disease				
No, n (%)	19 (95)	63 (86)	55 (92)	0.495^b^
Yes, n (%)	1 (5)	10 (14)	5 (8)	
Type 2 diabetes				
No, n (%)	19 (95)	69 (94)	55 (92)	0.898^b^
Yes, n (%)	1 (5)	4 (6)	5 (8)	
Tobacco use				
Active, n (%)	4 (20)	9 (12)	8 (13)	0.362^b^
Former, n (%)	10 (50)	26 (36)	28 (47)	
Never, n (%)	6 (30)	38 (52)	24 (40)	
Alcohol intake, median (IQR)	4.5 (1.0–6.5)	3.5 (1.0–7.0)	3.0 (2.0–8.5)	0.975^c^
BMI, mean (SD)	24.4 (7.4)	25.6 (5.2)	26.6 (5.1)	0.290^a^
CRP category				
< 3, n (%)	9 (56)	54 (87)	35 (75)	0.037^b^
3–10, n (%)	6 (38)	6 (10)	9 (19)	
10–15, n (%)	1 (6)	2 (3)	1 (2)	
> 15, n (%)	0 (0)	0 (0)	2 (4)	
CD4^+^ lymph, %, mean (SD)	41.0 (11.0)	48.2 (9.2)	47.6 (10.9)	0.018^a^
CD8^+^ lymph, %	23.8 (10.5)	22.4 (9.3)	20.7 (11.2)	0.428^a^
CD4/CD8 ratio	2.1 (1.1)	2.8 (1.9)	3.4 (2.8)	0.058^a^
Neutrophils, cells per µl	3.8 (1.3)	3.7 (1.3)	3.4 (1.1)	0.291^a^
Lymphocytes, cells per µl	1.8 (0.5)	1.8 (0.6)	1.8 (0.8)	0.890^a^
Neutrophil–lymphocyte-ratio	2.4 (1.3)	2.2 (1.1)	2.1 (0.9)	0.569^a^

^a^One-way ANOVA, ^b^Chi-square, ^c^Kruskal–Wallis.

### Does CFH polymorphism affect markers of inflammation and ageing in a population of healthy individuals?

**Figure 1:**
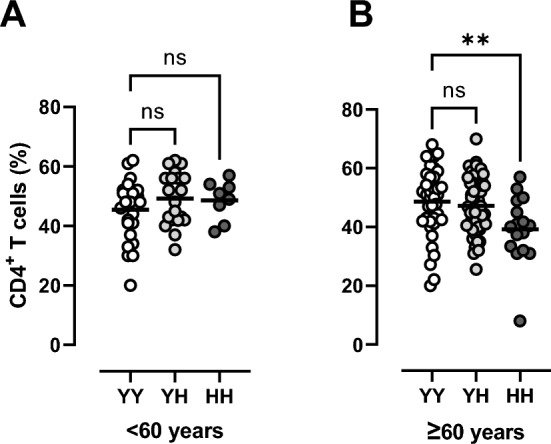
In healthy individuals with the HH genotype, we observed an age-related decline in proportion of CD4^+^ T cells, whereas participants with the YH or YY remained stable with increasing age. Asterisk denote P values according to one-way ANOVA with Dunnett’s multiple comparison test. **P < 0.01.

In the population of healthy individuals, the proportion of CD4^+^ T cells in individuals with the at-risk HH SNP was 41.0% (SD, 10.9), which was significantly lower than in individuals with the YY SNP 47.6% (11.0) (P = 0.022) and 48.2% (9.2) with YH SNP (P = 0.004) (Table 2). In a subgroup analyses based on age, it was seen that only individuals with the HH SNP aged 60 years or older demonstrated a lower proportion of CD4^+^ T cells (P < 0.01) (Fig. 1). Excluding the outlier in the group of HH SNP aged 60 years or older did not change the conclusion (P < 0.05, data not shown). None of the comorbidities or lifestyle choices were associated with the lower proportion of CD4^+^ T cells.

The proportion of CD8^+^ T cells was 22.6% (SD: 12.2) for all healthy individuals, and did not differ according to CFH SNP. Neither did the proportion of neutrophils and lymphocytes. Contrary, individuals with the HH SNP demonstrated elevated levels of CRP compared to individuals with the YY and YH SNPs (P = 0.037, Chi-square) (Table 2).

### Does CFH deficiency affect T cell distribution in a rodent model?

**Figure 2:**
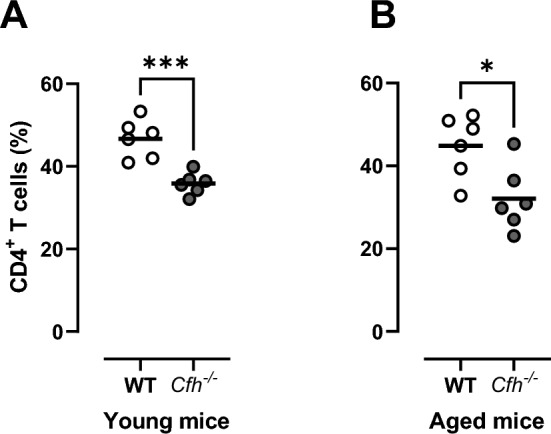
In mice with deficiency in *Cfh*, a decrease in the proportion of CD4 T cells was noted compared to wild type (WT). *P < 0.05; ***P < 0.001.

To explore the effect of decreased CFH function in an animal model, we used mice with complete deficiency in Cfh. Compared to wild type, we found lower proportions of CD4^+^ T cells in both the group of young (P < 0.001) and aged (P < 0.05) mice deficient in Cfh (Fig. 2).

### Does CFH polymorphism affect markers of inflammation and ageing in a population with dry AMD?

In the population of individuals with early and late dry AMD according to CARMS 2–4, the proportion of CD4^+^ T cells in individuals with the at-risk HH SNP was 39.9% (11.9), which was significantly lower compared to 46.4% (11.3) in individuals with the YY SNP (P = 0.046) and 47.5% (10.2) with YH SNP (P = 0.004). Contrary, the levels of CRP, neutrophils, and lymphocytes were similar across CFH genotypes in the group of participants with dry AMD (Table 3).

**Table 3:** Characteristics of the population of individuals with early and late dry AMD (CARMS 2–4) according to CFH Y402H genotype.

	CFH Y402H			P-value
	HH, N = 25	YH, N = 43	YY, N = 16	
Age, mean (SD)	76.6 (7.1)	78.9 (8.6)	79.8 (6.4)	0.377^a^
Gender				
F, n (%)	13 (52)	27 (63)	9 (56)	0.673^b^
M, n (%)	12 (48)	16 (37)	7 (44)	
Hypertension				
No, n (%)	10 (40)	23 (54)	10 (63)	0.339^b^
Yes, n (%)	15 (60)	20 (46)	6 (37)	
Hyperlipidemia				
No, n (%)	18 (72)	33 (77)	13 (81)	0.837^b^
Yes, n (%)	7 (28)	10 (23)	3 (19)	
Cardiovascular disease				
No, n (%)	19 (76)	32 (74)	9 (56)	0.379^b^
Yes, n (%)	6 (24)	11 (26)	7 (44)	
Type 2 diabetes				
No, n (%)	21 (84)	37 (86)	13 (81)	0.922^b^
Yes, n (%)	4 (16)	6 (14)	3 (19)	
Tobacco use				
Active, n (%)	10 (40)	9 (21)	3 (19)	0.532^b^
Former, n (%)	11 (40)	20 (47)	4 (25)	
Never, n (%)	4 (16)	14 (33)	9 (56)	
Alcohol intake, median (IQR)	7.0 (2.0–11.0)	3.0 (1.0–7.0)	2.0 (0.0–6.0)	0.184^c^
BMI, mean (SD)	27.5 (4.3)	26.5 (5.0)	24.4 (3.9)	0.128^a^
CRP category				
< 3, n (%)	14 (56)	25 (64)	9 (64)	0.974^b^
3–10, n (%)	9 (36)	10 (26)	4 (29)	
10–15, n (%)	1 (4)	2 (5)	1 (7)	
> 15, n (%)	1 (4)	2 (5)	0 (0)	
CD4^+^ lymph, %	39.9 (11.9)	47.5 (10.2)	46.4 (11.3)	0.024^a^
CD8^+^ lymph, %	27.1 (11.0)	24.9 (11.8)	26.0 (11.2)	0.751^a^
CD4/CD8 ratio	2.1 (2.2)	2.9 (2.8)	2.2 (1.2)	0.372^a^
Neutrophils, cells per µl	4.2 (1.5)	4.2 (1.7)	4.0 (1.9)	0.884^a^
Lymphocytes, cells per µl	1.6 (0.4)	1.5 (0.4)	1.6 (0.4)	0.817^a^
Neutrophil–lymphocyte-ratio	2.8 (1.2)	2.9 (1.3)	2.6 (1.1)	0.664^a^

^a^One-way ANOVA, ^b^Chi-square, ^c^Kruskal–Wallis.

### Does CFH polymorphism affect markers of inflammation and ageing in a population with neovascular AMD?

In the population of individuals with neovascular AMD (CARMS 5), the proportion of CD4^+^ T cells in individuals with the at-risk HH SNP was 47.5% (11.2), which did not differ from 51.8% (12.1) in individuals with the YY SNP (P = 0.199) and 44.6% (11.7) with YH SNP (P = 0.176). Similarly, no differences were noted in levels of CRP, neutrophils, or lymphocytes according to CFH SNP (Table 4).

**Table 4:** Characteristics of the population of individuals with neovascular AMD (CARMS 5) according to CFH Y402H genotype.

	CFH Y402H			P-value
	HH, N = 57	YH, N = 67	YY, N = 24	
Age, mean (SD)	74.1 (7.2)	76.9 (7.1)	77.9 (8.6)	0.046^a^
Gender				
F, n (%)	29 (51)	37 (60)	7 (29)	0.184^b^
M, n (%)	28 (49)	25 (40)	17 (71)	
Hypertension				
No, n (%)	30 (53)	34 (55)	12 (50)	0.926^b^
Yes, n (%)	27 (47)	28 (45)	12 (50)	
Hyperlipidemia				
No, n (%)	45 (79)	47 (76)	15 (63)	0.292^b^
Yes, n (%)	12 (21)	15 (24)	9 (37)	
Cardiovascular disease				
No, n (%)	45 (79)	53 (86)	19 (21)	0.597^b^
Yes, n (%)	12 (21)	9 (14)	5 (21)	
Type 2 diabetes				
No, n (%)	51 (89)	54 (87)	23 (96)	0.587^b^
Yes, n (%)	6 (11)	5 (13)	1 (4)	
Tobacco use				
Active, n (%)	14 (25)	16 (26)	6 (25)	0.258^b^
Former, n (%)	31 (54)	23 (37)	9 (38)	
Never, n (%)	12 (21)	23 (37)	9 (38)	
Alcohol intake, median (IQR)	6.0 (0.0–10.0)	5.0 (1.0–8.0)	2.0 (0.5–7.0)	0.573^c^
BMI, mean (SD)	26.1 (6.0)	25.1 (5.2)	25.3 (7.5)	0.653^b^
CRP category				
< 3, n (%)	25 (51)	32 (59)	15 (68)	0.698^b^
3–10, n (%)	16 (33)	18 (33)	5 (23)	
10–15, n (%)	4 (8)	1 (2)	1 (5)	
> 15, n (%)	4 (8)	3 (6)	1 (5)	
CD4^+^ lymph, %	47.5 (11.2)	44.6 (11.7)	51.8 (12.1)	0.061^a^
CD8^+^ lymph, %	22.9 (12.2)	24.0 (13.1)	17.5 (7.0)	0.141^a^
CD4/CD8 ratio	3.2 (2.9)	3.0 (2.8)	3.6 (2.9)	0.705^a^
Neutrophils, cells per µl	4.3 (1.5)	4.6 (1.4)	4.0 (0.8)	0.161^a^
Lymphocytes, cells per µl	1.6 (0.4)	1.6 (0.5)	1.8 (0.6)	0.288^a^
Neutrophil–lymphocyte-ratio	2.8 (1.1)	3.0 (1.2)	2.6 (1.4)	0.236^a^

^a^One-way ANOVA, ^b^Chi-square, ^c^Kruskal–Wallis.

## Discussion

We measured immunological alterations associated with the CFH Y402H polymorphism to assess the mechanistic link between CFH and development of AMD. We found that the HH polymorphism was associated with increased levels of CRP and with age a lower proportion of CD4^+^ T cells in healthy individuals without AMD. Using a mouse model, we found a similar decrease in levels of CD4^+^ T cells in mice with deficiency in Cfh.

The at risk HH SNP has previously been associated with increased levels of circulating proinflammatory cytokines including IL6 and CRP^22, 23^. To the best of our knowledge, this is the first study on the association between a CFH genotype and systemic levels of T cells. Few studies exist on the association between leukocytes and CFH genotypes. In the Rotterdam Study, it was reported that the CFH Y402H polymorphism had no effect on the white blood cell count^24^, which is in agreement with our findings.

CFH is a negative regulator of the complement system and CFH Y402H carriers have slightly increased activity in the complement system with increased levels of complement split-products in plasma^12–14^. These split-products may interact with T cells and professional antigen-presenting cells via membrane-bound receptors and lead to stronger T cell responses^15, 16^. In immune homeostasis, this may be a beneficial modulation of an immune response, but given complement dysregulation the abundance of complement split-products may evade the T cell regulation and lead to chronic inflammation^25^ and lower levels of CD4^+^ T cells^26^.

Complement factor H binds to CRP and thereby dampen its proinflammatory activity. This binding is impaired in the at-risk variant^27, 28^. As a consequence, individuals who carry the at-risk polymorphism have higher concentrations of CRP both in plasma^24, 28^ and in the RPE-choroid layer compared to those who are homozygous for the non-risk variant^29^. This corresponds with the finding that CRP was increased in healthy individuals with the at-risk variant in the present study.

The systemic nature of the changes and the finding that it is demonstrated in healthy individuals with the AMD-risk conferring genotype supports the notion that systemic proinflammatory stimulation of the retinal pigment epithelium (RPE) is a contributor to development of AMD. As such the presence of inflammatory mediators in the highly perfused choroid has previously been demonstrated to activate the RPE in both in vitro and in vivo studies^28, 30–32^.

Further, it has been previously been shown that patients treated for AIDS, demonstrated increased incidence of early development of AMD^33^. While immune-reconstituted HIV represents a very specialized form of immune deviation, it does demonstrate that immune dysregulation, low CD4 counts and systemic inflammation may promote development of AMD.

The immune alterations with CFH-genotypes differed in AMD-patients compared to healthy individuals. We found that only the patients with dry AMD displayed an association between low levels of CD4^+^ T cells and the CFH at-risk polymorphism. During the progression of AMD numerous changes occur, and several cross-sectional studies have reported on higher levels of proinflammatory markers in both dry and neovascular AMD^6, 11, 34^. Therefore, it seems intuitive that the proinflammatory changes associated with the at-risk CFH polymorphism HH is overshadowed by the presence of AMD and the associated immunological alterations. The ocular immune recruitment is probably more prominent in neovascular AMD than in dry AMD^35^, which may contribute to the increased levels of CD4^+^ T observed in patients with neovascular AMD in our study.

Several limitations should be kept in mind when interpreting these findings. In this study we did not include any individuals with any known immune disease, inflammatory disorder, cancer, or persons who received immune modulating medication. Therefore, our results can only be generalized to this group of persons. Because complement factor H polymorphism and low CD4 count are both associated with autoimmune inflammatory disorders^15, 26^, this could potentially lead to a selection bias. Another limitation is the relative low numbers of patients with early dry AMD, which hinders subgroup analysis of patients with dry AMD. A large-scale study could validate our findings.

Further, CD4^+^ T cells are a group of heterogenous immune cells, and our data does not provide any insight into the differentiation and properties of these immune cells. Additional studies could examine possible functional or phenotypical differences in the CD4^+^ T cells according to CFH genotype.

In mice deficient in Cfh, we found lower proportions of CD4^+^ T cells in both young and aged mice. The rodent model of Cfh-deficiency is not identical to the decreased complement regulation with CFH Y402H polymorphisms. The Cfh deficient mice have complete loss of Cfh resulting in undetectable levels of plasma C3^20^. This may explain why no age-effect is observed in mice and also demonstrate that the loss of complement regulation has an influence on the distribution of CD4^+^ T cells thereby strengthening our results.

It is well-known that carriers of the CFH Y402H have increased risk of AMD^3, 24^. An implication of our study is that these individuals demonstrate increased systemic inflammation at a stage before signs of AMD can be detected clinically. Thus, if an anti-inflammatory prevention/treatment should be available, this should be administered to individuals based on risk of disease rather than signs of disease.

In conclusion, we found that healthy individuals with the AMD-risk conferring CFH Y402H genotype displayed decreased levels of CD4^+^ T cells and increased levels of CRP in plasma. Mice with deficiency in Cfh also displayed decreased levels of CD4^+^ T cells. This indicates that decreased complement regulation results in extensive changes in innate and adaptive immune compartments that precede development of AMD.

## Data Availability

The datasets used during the current study are available from the corresponding author on reasonable request.
